# Liquid–Liquid Equilibrium of Sesame Fatty Acid (Ethyl and Methyl) Ester + Glycerol + Ethanol/Methanol Mixtures at Different Temperatures

**DOI:** 10.3390/molecules29133167

**Published:** 2024-07-03

**Authors:** Anderson Silva, Guilherme Lopes, Marcos Corazza, Pedro Arce, Dayana Coêlho, Lucas Meili, Sandra Carvalho, Leandro Ferreira-Pinto, João Soletti

**Affiliations:** 1 Laboratory of Separation Systems and Process Optimization (LASSOP)-Center of Technology, Federal University of Alagoas, Maceió 57072-900, AL, Brazil; anderson.engquimica@yahoo.com.br (A.S.); dayana.coelho@ctec.ufal.br (D.C.); lucas.meili@ctec.ufal.br (L.M.); sandra.ufal@gmail.com (S.C.); jisoletti@gmail.com (J.S.); 2 Department of Engineering, School of Engineering and Sciences, Sao Paulo State University (UNESP), Rosana 19274-000, SP, Brazil; guilherme.s.lopes@unesp.br; 3 Department of Chemical Engineering, Federal University of Parana, Curitiba 80060-000, PR, Brazil; corazza@ufpr.br; 4 Department of Chemical Engineering, Engineering School of Lorena (EEL/USP), University of Sao Paulo, Lorena 12602-810, SP, Brazil; parce@usp.br

**Keywords:** sesame oil, biodiesel, alcohol, liquid–liquid equilibrium, NRTL

## Abstract

This study aimed to investigate the liquid–liquid equilibrium (LLE) behavior of sesame fatty acid ethyl ester (FAEE) and methyl ester (FAME) in combination with glycerol and the co-solvents ethanol and methanol. FAEE and FAME were produced through the transesterification of mechanically extracted and purified sesame oil, using potassium hydroxide (KOH) as a homogeneous base catalyst. The reactions were conducted in ethanol and methanol to produce FAEE and FAME, respectively. Post-reaction, the products were separated and purified, followed by an analysis of the LLE behavior at 313.15 K and 323.15 K under atmospheric pressure (101.3 kPa). The experimental process for the miscibility analysis utilized a jacketed glass cell adapted for this study. Miscibility limits or binodal curves were determined using the turbidity-point method. Tie lines were constructed by preparing mixtures of known concentrations within the two-phase region, which allowed the phases to separate after agitation. Samples from both phases were analyzed to determine their composition. This study revealed that higher temperatures promoted greater phase separation and enhanced the biodiesel purification process. The NRTL model effectively correlated the activity coefficients with the experimental data, showing good agreement, with a root-mean-square deviation of 3.5%. Additionally, the data quality was validated using Marcilla’s method, which yielded an R^2^ value close to 1. Attraction factors and distribution coefficients were also calculated to evaluate the efficiency of the co-solvents as extraction agents. The findings indicated higher selectivity for methanol than for ethanol, with varying degrees of distribution among the co-solvents. These results offer significant insights into enhancing biodiesel production processes by considering the effects of co-solvents on the LLE properties of mixtures, ultimately contributing to more efficient and cost-effective biodiesel production.

## 1. Introduction

Biodiesel is a mixture of esters derived from animal or vegetable oils and is primarily obtained through the transesterification or esterification of fatty acids. This process typically involves using an excess of alcohol to shift the reaction towards product formation and requires an alkaline catalyst to enhance the reaction speed. Methanol is commonly used because of its low cost and favorable physicochemical characteristics. However, ethanol is increasingly preferred in Brazil because of its availability, low toxicity, high dissolving capability, and carbon neutrality, as it is derived from plants. Brazil’s diverse climate and soil conditions have significant potential for producing biodiesel from various oleaginous plants, such as soy, palm, peanut, and sesame [1,2,3,4,5].

Sesame (*Sesamum indicum* L.) is one of the world’s oldest known oilseeds, indigenous to the African continent and widely cultivated across the tropics and subtropics [6,7]. It matures within three–four months and is drought-tolerant, requiring minimal rainfall to thrive [8]. Ideal climatic conditions for sesame production in Brazil are found in the semiarid northeast region. The relative humidity of the air, typically 60%, and continuous solar radiation lead to low disease incidence, increased plant growth, and production of high-quality seeds [9]. Sesame seeds contain a high concentration of nutritional components and oil content (46–50%) and saturated fatty acids (80–90%), making them attractive for biodiesel production due to their functional and nutraceutical properties [10,11]

According to Egbekun et al. [12], variations in sesame seed oil yield can be attributed to differences in genotypes, climatic conditions, plant maturation stages, harvest times, and extraction methods used. Antoniassi et al. [13] report in their study that the fatty acid profile of sesame seed oil differs for seeds harvested in different climates. Arslan et al. [14] state that the oleic/linoleic acid ratio is strongly influenced by external factors.

Sesame oil is a promising alternative feedstock for biodiesel production, capable of diversifying the sources of raw materials and reducing the heavy reliance on soybean oil. Embrapa (Empresa Brasileira de Pesquisa Agropecuária) has developed high-yield, disease-resistant sesame cultivars such as G4 and BRS Seda, which are adapted to various Brazilian regions. These developments have enhanced the competitiveness of sesame as a biodiesel feedstock, contributing to more sustainable and resilient biofuel production systems [13,15].

The efficiency of biodiesel production relies heavily on the effective separation and purification of the reaction products, particularly biodiesel, glycerol, and alcohols. Understanding the phase diagrams of these multicomponent mixtures is crucial for optimizing the production process [16,17]. Determining the miscibility and immiscibility regions allows for an accurate assessment of the relative amounts of biodiesel, glycerol, and ethanol, which aids in optimizing the production process, reducing operational costs, and improving biodiesel quality. However, there is a notable lack of studies on the liquid–liquid phase equilibrium of sesame biodiesel systems, highlighting the need for further research [16,17].

The purpose of this study was to determine the equilibrium (liquid + liquid) and solubility curves at 313.15 and 323.15 K at atmospheric pressure (101.3 kPa, Maceió City, State of Alagoas, Brazil) and the respective tie lines for the systems {fatty acid ethyl ester (FAEE) + glycerol + ethanol} and {fatty acid methyl ester (FAME) + glycerol + methanol}. Fatty acid ethyl esters (FAEEs) and fatty acid methyl esters (FAMEs) were produced through the transesterification of sesame oil using sodium hydroxide as a catalyst. The NRTL model was used to correlate the activity coefficients with the experimental data. Hand and Othmer–Tobias correlations were used to assess the data quality and establish tie lines. These studies are not usually found in the literature and constitute an original contribution.

## 2. Experimental

All the analytical and experimental methodologies utilized in this study have been previously published. Several research groups have discussed these techniques [18]. However, in the current work, only analytical and experimental methodologies are presented in minor detail.

### 2.1. Materials

The sesame seeds used in this study were obtained from a prominent market in Maceió, AL (Brazil). The seeds were dried in a vented oven at 333.15 K for 24 h prior to the experiments. The oil was extracted using a hydraulic press (TECNAL^®^-TE098, Piracicaba, Brazil) under 12 tons of pressure, filtered (porosity of 2 m), and dehydrated using magnesium sulfate [18] (>97 wt%, acquired from Nuclear^®^, Rio de Janeiro, Brazil) at 333.15 K. Anhydrous ethanol (>99 wt%), methanol (>99 wt%), glycerol (>99 wt%), and sodium hydroxide (>97 wt%) were obtained from Nuclear^®^ (Brazil) and used without prior purification.

### 2.2. Production of Methyl and Ethyl Biodiesel from Sesame Oil (Sesamum indicum)

The production methodology was based on the specialist literature [18,19] and is briefly discussed in this text.

The pilot module comprised a 2 L encased glass reactor, a thermostated bath (TECNAL, TE184), and a mechanical stirrer (TECNAL, TE2003). The pilot modulo was agitated continuously and maintained at 323.15 K. After stabilizing the experimental conditions, the reactor was filled with potassium hydroxide (1 wt%) and alcohol. The reaction was conducted at an oil–alcohol molar ratio of 1:10 (oil–alcohol) for 30 min. After the completion of the reaction, the product was collected and centrifuged to separate the biodiesel- and glycerol-rich phases.

FAEEs and FAMEs were washed repeatedly with distilled water until the pH reached 7.0, with a progressive reduction in washing water volume. Initially, a water–biodiesel ratio of 1:10 at 343.15 K was employed to remove the catalyst, ethanol, and mono- and diacylglycerols. The pH was adjusted by adding sulfuric acid solution at a specific mass ratio of sulfuric acid to biodiesel. After each wash, phase separation was achieved via centrifugation, and the pH of the biodiesel-rich phase was verified. Subsequent washes with sterile water were conducted until a pH of 7.0 was reached. The purified biodiesel was then dried with manganese sulfate (MnSO_4_) and filtered, and the yield of the transesterification/esterification reaction was determined with gas chromatography to quantify the total esters [18].

A GC-2010/Shimadzu instrument (Shimadzu, San Jose, CA, USA) equipped with a split/splitless injection system was used, operating at 523.15 K, with a split ratio of 100:1, sample injection volume of 1.0 μL, and flame ionization detector (FID) operating at 523.15 K. A polar capillary column ZB-WAX plus/Phenomenex (Torrance, CA, USA) was employed, measuring 30 m in length, 0.32 mm in internal diameter, with a film thickness of 0.25 μm. High-purity hydrogen gas (99.95% LINDE) was used as the carrier gas. The temperature program for the oven and column was as follows: 433.15–498.15 K at 15 K/min, then 433.15–498.15 K at 3 K/min, resulting in a total analysis time of 11 min. The fatty acid composition was determined by identifying fatty acids by comparing retention times with those of standard ester mixtures (tricaprylin). Equation (1) was used to integrate the peak areas via normalization to quantify the fatty acids. The chemical composition of the major fatty acids was determined via gas chromatography (GC) using the European Standard EN14103 [20] (see Table 1).

(1) $$\%yield=\frac{m_{tricaprylin}A_{B}f_{tricaprylin}}{A_{tricaprylin}m_{s}}\cdot 100$$

where m_tricaprylin_, f_tricaprylin_, and A_tricaprylin_ represent the mass, response factor, and peak area of the internal standard, respectively; A_B_ is the sum of the peak areas of FAMEs or FAEEs; and m_s_ is the mass of the sample.

The average molar masses of sesame FAEEs and FAMEs were calculated based on the fatty acids listed in Table 1, yielding values of 307.738 and 293.714 g/mol for FAEEs and FAMEs, respectively. To estimate the molar mass of the esters formed, we assumed that each fatty acid was completely converted into its respective ester during the transesterification reaction. Based on this assumption, we utilized the methodology described by Halvorsen et al. [21] to estimate the molecular weights of the produced esters.

**Table 1:** Major fatty acid components of sesame oil used in this study.

Compound	Fatty Acid	Cx:y ^a^	Molar Mass (g. gmol^−1^)	Content 100.w			
				This Study ^b^	Carvalho et al. [22]	Corso et al. [23]	Andresa et al. [24]
**1**	Palmitic acid	16:0–C_16_H_32_O_2_	256.43	10.5	11.5	10.3	9.7
**2**	Stearic acid	18:0–C_18_H_36_O_2_	284.48	2.8	5.3	5.2	6
**3**	Oleic acid	18:1–C_18_H_34_O_2_	282.46	35.8	38.1	36.4	38.7
**4**	Linoleic acid	18:2–C_18_H_32_O_2_	280.45	50.9	43.1	46.8	45

^a^ Cx:y, x = carbon number, y = number of double bonds. ^b^ Standard uncertainties *u* (wt%) = ±0.6 wt%.

### 2.3. Apparatus and Procedures for Equilibrium Liquid–Liquid

#### Liquid–Liquid Equilibrium Data

**Figure 1:**
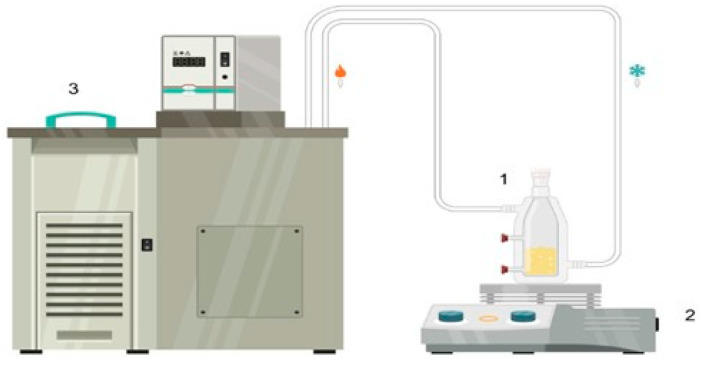
Experimental apparatus of the equilibrium cell: (1) cell Equilibrium; (2) magnetic stirrer bar; (3) thermostatic bath.

The literature [17,25] provides an approach for determining binodal and tie line curves. A turbidimetric study employing a titration technique under isothermal conditions was conducted to establish the phase boundaries. A jacketed equilibrium cell with a capacity of 50 mL (see Figure 1) was used, which was coupled to a thermostatic bath (TECNAL, TE18, with a temperature uncertainty of ±0.5 K) set at 313.15 and 323.15 K. The temperature controller of the thermostatic bath, connected to a thermocouple, was calibrated using a primary thermometer to ensure the precision of the experimental temperatures. A magnetic stirrer (PHOX, MS-HS2, HCS Scientific & Chemical Pte Ltd., Singapore) was used to agitate the mixture.

To establish tie lines, known quantities of glycerol and biodiesel were initially introduced into the equilibrium cell (keeping the same mass ratio for every experiment). This combination was then added to ethanol or methanol to achieve different overall compositions and, as a result, distinct tie lines. The preset mixtures were stirred for 12 h using a mechanical stirrer. Following this procedure, two phases with well-defined interfaces were formed. Samples from each phase were collected for further analyses. To evaluate the ethanol or methanol composition of the ternary system, the extract and raffinate were evaporated to a constant weight in a drying oven at 343.15 K (to a stable weight) to determine the mass fraction of alcohol. The tie lines were constructed by interpolating the viscosity values measured on and below the binodal curve (Ostwald viscometer with a 300 cSt/s capillary, VWR, Radnor, PA, USA).

The mixture composition was calculated using the previously measured binodal curves by determining the point reflecting the content of the specified component. At least three independent measurements were performed for each sample and phase with an average uncertainty of less than 1 wt%. Mass balances were verified using the total system mass, phase, and overall composition.

### 2.4. Quality Test of the Experimental Data

According to Marcilla et al. [26], the LLE experimental data can be evaluated based on the mass balance; that is, the sum of the masses calculated in both phases is compared with the real value of the total mass used in the experiment. The mass balance of each component of the ternary system can be calculated using Equations (2)–(4).

(2) $$W^{sol}w_{1}^{sol}=W^{L1}w_{1}^{L1}+W^{L2}w_{1}^{L2}$$

(3) $$W^{sol}w_{2}^{sol}=W^{L1}w_{2}^{L1}+W^{L2}w_{2}^{L2}$$

(4) $$W^{sol}w_{3}^{sol}=W^{L1}w_{3}^{L1}+W^{L2}w_{3}^{L2}$$

where w_1_^L1^, w_1_^L2^; w_2_^L1^, w_2_^L2^; and w_3_^L1^, w_3_^L2^ represent the compositions (mass fractions) of components 1, 2, and 3 of the liquid phases, L1 and L2, at equilibrium. The system of linear algebraic equations (Equations (2)–(4)) can be represented in matrix terms as follows (Equation (5)).

(5) $$\underset{Y}{\underbrace{\left[\begin{array}{c} W^{sol}w_{1}^{sol}\\ W^{sol}w_{2}^{sol}\\ W^{sol}w_{3}^{sol} \end{array}\right]}}=\underset{Z}{\underbrace{\left[\begin{array}{cc} w_{1}^{L1} & w_{1}^{L2}\\ w_{2}^{L1} & w_{2}^{L2}\\ w_{3}^{L1} & w_{3}^{L2} \end{array}\right]}}.\underset{P}{\underbrace{\left[\begin{array}{c} W^{L1}\\ W^{L2} \end{array}\right]}}$$

By exploiting the vector P, it can be expressed as (Equation (6)):

(6) $$P={\left(Z^{T}Z\right)}^{-1}Z^{T}Y$$

where ZT represents the transposed matrix of Z and (Z^T^ Z)^(−1)^ represents the inverse of the matrix (Z^T^ Z). 

By solving Equation (7) numerically, the values of W^L1^ and W^L2^ can be obtained, and the sum of W^L1^ and W^L2^ can be compared with the value of W^sol^. The general deviation of the mass balance (δ) can be obtained as follows:

(7) $$\delta \left(\%\right)=100\cdot \frac{\left|\left(w^{L1}+w^{L2}\right)-w^{sol}\right|}{w^{sol}}$$

According to Marcilla et al. [26], general deviations of the mass balance, δ, of less than 2.0% guarantee a good quality of the experimental data.

### 2.5. Distribution Coefficient and Selectivity

The distribution coefficient (*D*_2_) and separation factor (*S*) were calculated from the LLE data for the ternary systems. These parameters are defined as follows (Equations (8) and (9)).

(8) $$D_{2}=\frac{w_{2}^{II}}{w_{2}^{I}}$$

(9) $$S=\frac{\left(w_{2}^{II}/w_{2}^{I}\right)}{\left(w_{1}^{II}/w_{1}^{I}\right)}$$

where $w_{2}^{I}$ and $w_{2}^{II}$ represent the mass fractions of alcohol in the FAME or FAEE and glycerol phases, respectively; $w_{1}^{I}$ and $w_{1}^{II}$ represent the mass fractions of FAME or FAEE in the FAME or FAEE and glycerol phases [27]. 

## 3. Thermodynamic Modeling

Ferrari et al. [28] proposed an approach to LLE computation and parameter estimation. The liquid–liquid equilibrium was calculated using the multiphase liquid–liquid flash method and phase stability test. The activity coefficients were calculated using the NRTL model. 

The Non-Random Two-Liquid (NRTL) model [29] (Equation (10)) was adopted to correlate the measured LLE data from the tie line. The activity coefficient calculated using the NRTL model is

(10) $$ln\gamma_{1}=\left(\frac{\sum_{j=1}^{n}\tau_{ji}x_{j}G_{ji}}{\sum_{k=1}^{n}x_{k}G_{ki}}\;\right)+\sum_{j=1}^{n}\left(\frac{x_{j}G_{ij}}{\sum_{k=1}^{n}x_{k}G_{kj}}\right)\left(\tau_{ij}-\frac{\sum_{m=1}^{n}\tau_{mi}x_{m}G_{mi}}{\sum_{k=1}^{n}x_{k}G_{kj}}\right)$$

In which *x_i_* denotes the mole fraction of component *i*, γ_i_ is the activity coefficient of component *i*, and *T* is the experimental temperature. The interaction parameters are described by Equation (11):

(11) $$\tau_{ij}=\frac{g_{ji}-g_{ii}}{RT}=\frac{∆g_{ji}}{T}\;\;\;\;\;\;\;\;\;\;\;\;G_{ij}=exp\left(-\propto_{ij}\tau_{ij}\right)$$

where g_ij_ is the binary interaction parameter and α_ij_ is a non-randomness factor.

The binary interaction parameter for the thermodynamic model was optimized using a weighted least-squares objective function, as shown in Equation (12):

(12) $$minFO=\sum_{k=1}^{NP}\;\sum_{j=1}^{nf}\;\sum_{i=1}^{nc}\;\frac{{\left(x_{ijk}^{calc}-x_{ijk}^{exp}\right)}^{2}}{\sigma_{j}^{2}}$$

where $x_{ijk}^{calc}$ and $x_{ijk}^{\exp}$ are the calculated and experimental molar fractions of component i in phase j for the k tie line. *NP* is the total number of tie lines, *nf* is the total number of phases, *nc* is the number of compounds in the system, and $\sigma_{j}^{2}$ is the variance of the experimental phase.

The objective function minimization (Equation (12)) was performed using the Particle Swarm Optimization algorithm [28] as an initial estimate and the modified simplex method [30] as the final value of the binary parameter optimization.

To determine the connection between the NRTL model and experimental data, we used Equations (13) and (14) to obtain the root-mean-square deviation (rmsd%) and absolute deviation (AD%):

(13) $$rmsd=100\sqrt{\frac{\sum_{k=1}^{NP}\;\sum_{j=1}^{nf}\;\sum_{i=1}^{nc}\;{\left(x_{ijk}^{calc}-x_{ijk}^{exp}\right)}^{2}}{NP\times nf\times nc}}$$

(14) $$AD=100\frac{\sum_{k=1}^{NP}\;\sum_{j=1}^{nf}\;\sum_{i=1}^{nc}\;\left|x_{ijk}^{\mathrm{calc}}-x_{ijk}^{exp}\right|}{NP\times nf\times nc}$$

The obtained tie lines were correlated with the binary parameters of the NRTL model, considering the interaction between sesame oil, FAEE/FAME, glycerol, and alcohol (ethanol or methanol). The interactions between these components were correlated with the binary parameters of the NRTL model using the obtained tie lines.

## 4. Results and Discussions

### 4.1. Experimental Data

The experimental ternary LLE data for the system {FAEE or FAME + glycerol + alcohol} at temperatures of (313.15 and 323.15) K and atmospheric pressure (101.3 kPa) are provided in Table 2 and Table 3 (ethanol) and Table 4 and Table 5 (methanol).

The experimental results were assessed for correctness and precision using type-A uncertainty computed using the standard deviations of the analytical measurements [31]. Uncertainties in the composition of the equilibria varied from (0.03 to 0.5)% by mass for fatty acid esters, (0.06–0.9)% for alcohols, and (0.05–0.7)% for glycerol.

### 4.2. Quality Test of the LLE Data

Before submitting the LLE experimental data to thermodynamic modeling, it was necessary to analyze the quality of the data.

Once thermodynamic equilibrium was reached, the tie line compositions were w_1_^Bio^, w_2_^Bio^ e w_3_^Bio^ e w_1_^Gly^, w_2_^Gly^ e w_3_^Gly^, and W^Sol^ e w_1_^Sol^, w_2_^Sol^, and w_3_^Sol^. 

Using Equation (6), W^Bio^ and W^Gly^ were determined using numerical techniques to solve a system of three linear algebraic equations [26]. Once the W^Bio^ and W^Gly^ values were obtained, W^sol(calc)^ was calculated using a simple sum (mass balance). Thus, W^sol^ and W^sol(calc)^ were compared using Equation (7) to calculate the general mass balance deviation (δ). The results for the FAEE or FAME + glycerol + alcohol systems are presented in Table 6 and Table 7 for the FAEE + glycerol + ethanol and FAME + glycerol + methanol systems, respectively, at a temperature of 298.15 K.

From the results in Table 6 and Table 7, it can be observed that all individual deviations of each tie line were less than 2.0% for the FAEE or FAME + glycerol + alcohol systems, as recommended by the method proposed by Marcilla et al. [26], highlighting that all tie lines showed deviations lower than 0.50%. The average general deviation was 0.0848% and 0.1910% for the FAEE + glycerol + ethanol and FAME + glycerol + methanol systems, respectively, at a temperature of 298.15 K. From the deviations obtained, at a temperature of 298.15 K, it can be deduced that the experimental data for both systems are of good quality.

### 4.3. Separation Factor and Distribution Coefficient

The experimental values of the distribution coefficient and separation factor for the FAEE + glycerol + ethanol and FAME + glycerol + methanol systems are listed in Table 8 and Table 9, respectively. A system separation factor greater than one (S > 1) for both temperatures indicates that it is possible to extract the alcohol (solute) contained in FAEE or FAME (diluent) using glycerol (solvent).

**Figure 2:**
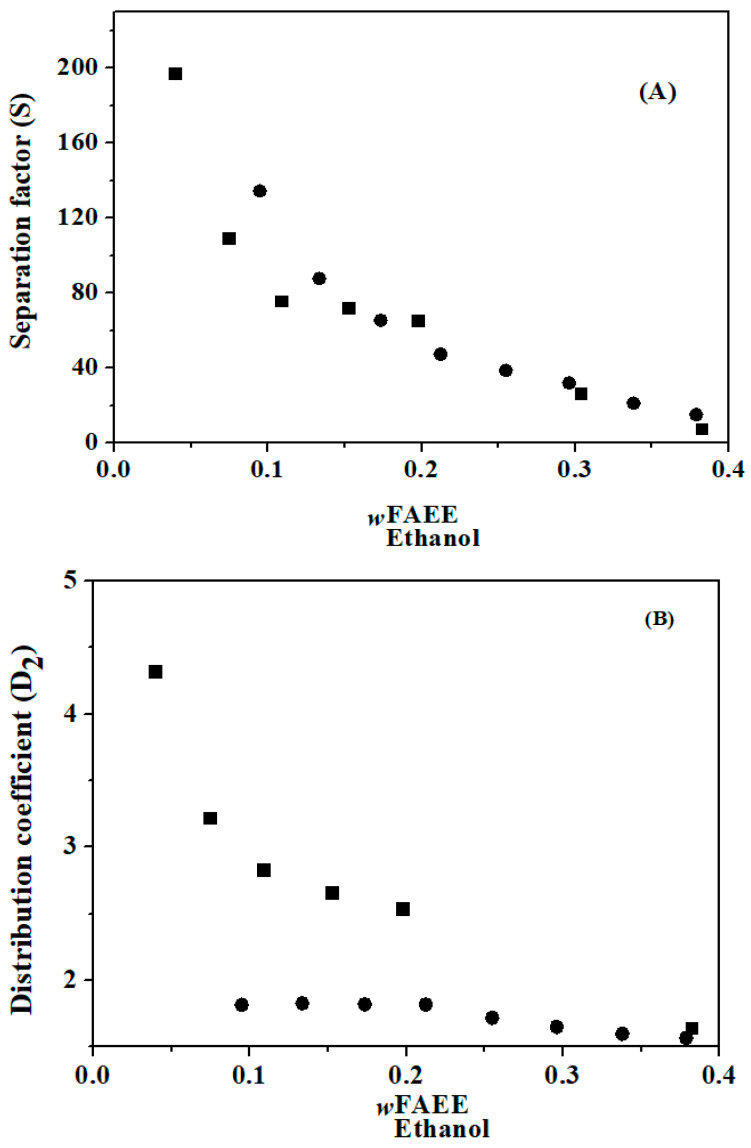
Separation factor (**A**) and distribution coefficient (**B**) for the {FAEE (1) + glycerol (2) + ethanol (3)} system (■, 313.15 K and 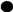, 323.15 K).

**Figure 3:**
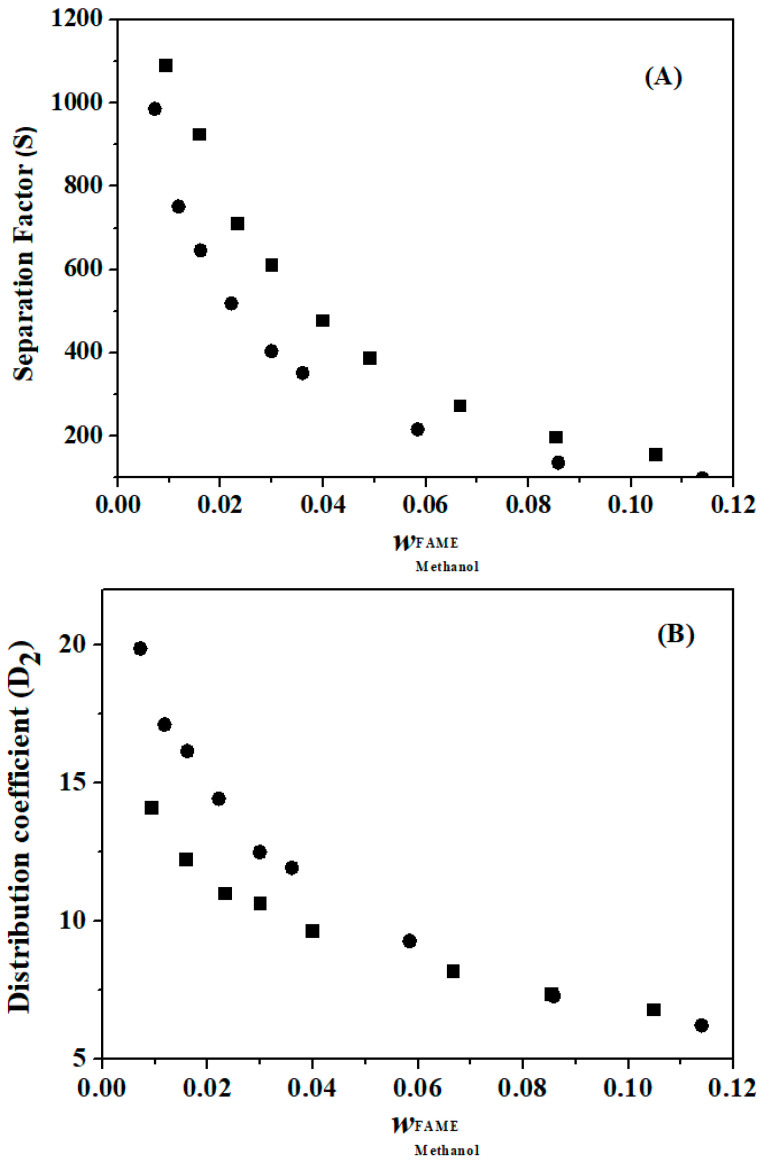
Separation factor (**A**) and distribution coefficient (**B**) for the {FAME (1) + glycerol (2) + methanol (3)} system (■, 313.15 K and 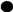, 323.15 K).

The values of S and D_2_ are presented in Figure 2 and Figure 3 for the FAEE + glycerol + ethanol and FAME + glycerol + methanol systems, respectively, as functions of the mass fraction of alcohol in the biodiesel phase (w_2_ diluent phase), which is rich in alcohol. In Figure 2, it is noted that all S values are greater than 1.0, indicating that the extraction of ethanol from the FAEE + glycerol solution is possible using glycerol as the extracting agent. In the same figure, the distribution coefficients (D_2_) for this system were greater than 1.0. Similar behavior was observed for the FAME + glycerol + methanol system (Figure 3) for the distribution coefficient and separation factor. Methanol can be extracted using glycerol, which acts as an extracting agent, owing to values greater than 1 in the separation factors.

### 4.4. Thermodynamic Modeling

**Figure 4:**
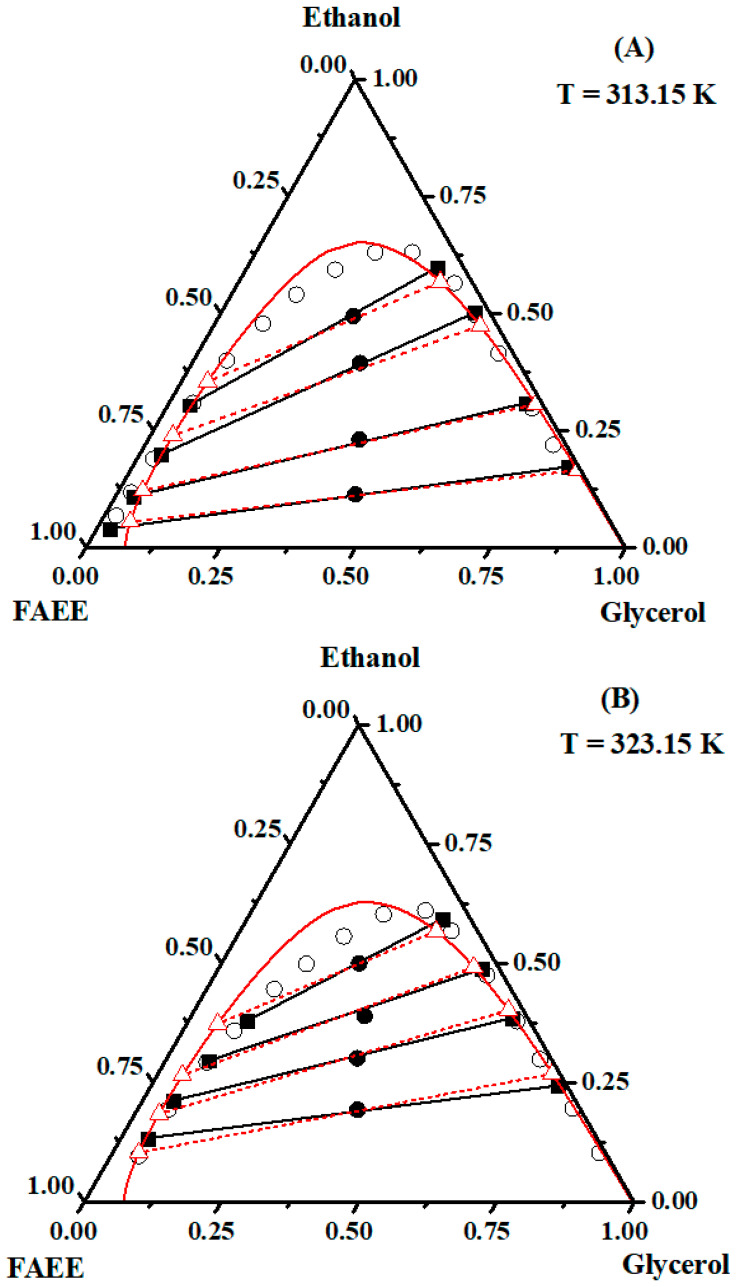
Ternary diagram (mass fraction) of the system {FAEE (1) + glycerol (2) + ethanol (3)} at (**A**) 313.15 K and (**B**) 323.15 K. Experimental (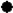, overall composition; 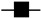, tie line; and 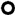, binodal points) and NRTL model (△
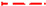
△, tie line; and 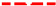, binodal line).

**Figure 5:**
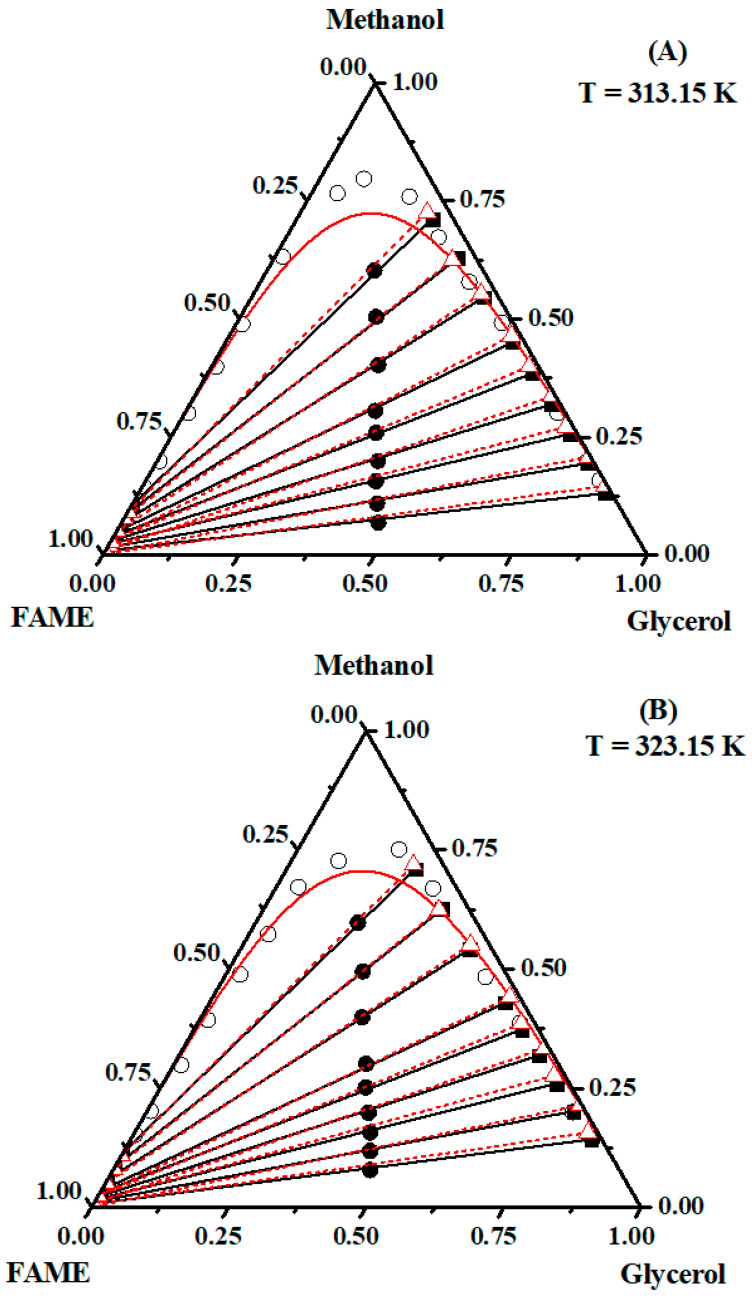
Ternary diagram (mass fraction) of the system {FAME (1) + glycerol (2) + ethanol (3)} at (**A**) 313.15 K and (**B**) 323.15 K. Experimental (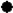, overall composition; 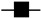, tie line; and 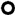, binodal points) and NRTL model (△
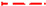
△, tie line; and 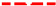, binodal line).

The tie lines obtained for the systems at 313.15 and 323.15 K showed a characteristic behavior, with an inclination angle varying according to the distribution of the ethanol or methanol in the rich phase (FAEE/FAME or glycerol). At 313.15 and 323.15 K, there was a slight increase in the immiscibility region (below the curve), indicating that phase separation favored sesame FAEE or FAME production. The quantitative analysis of the products present in the equilibrium phases aided the tie line construction. Points below the equilibrium curve (where the two phases existed) were used, and each phase corresponded to the ends of the tie lines (Figure 4 and Figure 5 for ethanol and methanol, respectively). 

Increasing the temperature of the system by 10 K (from 313.15 to 323.15 K) affected the interaction of the glycerol/ethanol and glycerol/methanol pairs. At 323.15 K, the phases were more separated; hence, the purification of sesame oil FAEE or FAME was easier because of the greater pair affinity.

The tie lines show that the curves constructed for the systems containing FAEE or FAME/glycerol/alcohol have good slopes. These tie lines reflect the overall composition of points. Temperature rises from 313.15 and 323.15 K slightly influenced the inclination of the tie lines in the ternary diagrams of the systems biodiesel/glycerol/alcohol. It is noted from these figures that in all cases, good agreement was verified between the calculated tie line values from the NRTL model and the experimental values. The NRTL model accurately correlated with the experimental data, as indicated by the root-mean-square deviation of the order of 1% of the binary interaction parameters, as shown in Table 10.

From the results of the separation factors, it was demonstrated that glycerol, acting as a solvent, is the best extraction agent for methanol and ethanol compared to biodiesel (separation factors well above 1.0). Methanol and ethanol are more soluble to a greater extent in glycerol than in biodiesel, and glycerol is also known to be insoluble in biodiesel. This can be explained via the mechanism proposed by Zhang and Wu [32]. In the mechanism responsible for the insolubility of glycerol in biodiesel in the absence of methanol, glycerol molecules tend to attract each other rather than disperse in the biodiesel structure, probably because of the entangled structure of glycerol and the network structure of biodiesel. The strong intermolecular force (hydrogen bonding) of glycerol and its elongated shape causes the glycerol to be entangled and, therefore, difficult to disperse unless an appropriate number of compound(s) with comparable intermolecular strength can be involved. Although it is possible that some components of biodiesel (such as water) are individually soluble in glycerol, such accessibility may be low because of the network structure formed by heavy compounds (such as oligomers) in biodiesel. On the other hand, methanol or ethanol, as amphiphilic compounds, can help in the dispersion of glycerol. Therefore, a homogeneous mixture was formulated and prepared with the addition of appropriate amounts of methanol or ethanol.

## 5. Conclusions

This study reports on liquid–liquid equilibrium (LLE) diagrams for ternary systems consisting of {FAEE or FAME + glycerol + ethanol or methanol} at temperatures of 313.15 and 323.15 K. The data included mutual solubility (binodal curves), tie lines, and overall mixture compositions. Correlations using Marcilla’s method demonstrated that the experimental tie line data are coherent and reliable. Furthermore, the NRTL activity coefficient model was used to correlate the experimental data with the binary interaction parameters for each binary interaction. The NRTL model effectively represented the experimental values for these highly non-ideal mixtures with a root-mean-square deviation of 3.5%. These findings indicate that glycerol, acting as a solvent, is a more efficient extraction agent for methanol and ethanol than biodiesel, as evidenced by separation factors well above 1.0. The strong intermolecular forces in glycerol, combined with its elongated shape, result in a tendency for glycerol molecules to attract each other rather than disperse in the biodiesel. However, the amphiphilic nature of methanol and ethanol aids in dispersing glycerol and forming homogeneous mixtures when added in appropriate amounts. These results offer significant insights into the LLE behavior of the {FAEE (1) + glycerol (2) + ethanol (3)} and {FAME (1) + glycerol (2) + methanol (3)} systems. They provide valuable information for optimizing biodiesel production processes by reducing operating costs and enhancing biodiesel quality. The conclusions of this study contribute to the broader field of biodiesel research by highlighting the importance of co-solvents in improving the efficiency and effectiveness of biodiesel purification and separation processes.

## Figures and Tables

**Table 2:** Binodal curve data of the ternary system {FAEE (1) + glycerol (2) + ethanol (3)} at 313.15 K and 323.15 K at normal atmospheric pressure (101.3 kPa), where *w* is the weight fraction ^a^.

T = 313.15 K			T = 323.15 K		
100.w_1_	100.w_2_	100.w_3_	100.w_1_	100.w_2_	100.w_3_
90.91	2.35	6.74	85.20	5.20	9.60
85.66	2.60	11.74	74.90	5.70	19.40
77.99	3.12	18.89	63.00	7.70	29.30
64.60	4.52	30.88	54.70	9.40	35.90
53.86	6.20	39.94	43.10	12.30	44.60
43.21	8.96	47.83	34.60	15.50	49.90
33.97	11.97	54.06	24.90	19.40	55.70
24.07	16.54	59.39	15.40	24.30	60.30
14.83	22.1	63.07	7.40	31.50	61.10
8.00	28.90	63.10	4.80	38.40	56.80
3.50	40.00	56.50	3.00	49.50	47.50
3.10	47.16	49.74	2.40	59.70	37.90
2.82	55.67	41.51	2.20	67.9	29.90
2.52	67.75	29.73	1.40	79.00	19.60
2.48	75.62	21.90	1.20	88.50	10.30

An estimated standard error of 1% was used. ^a^ Standard uncertainties *u* are *u*(T) = 0.5 K, *u*(*P*) = 1 kPa, *u*(*w*) ≤ 1.0 wt%. w_1_: mass FAEE; w_2_: mass fraction glycerol; w_3_: mass fraction ethanol.

**Table 3:** The phase equilibrium data (liquid + liquid) for the system {FAEE (1) + glycerol (2) + ethanol (3)} at 313.15 K and 323.15 K at normal atmospheric pressure (101.3 kPa), where *w* is the weight fraction ^a^.

T (K)	Overall Composition (Sol)			Experimental (Tie Lines)					
				Biodiesel Rich-Phase (FAEE)			Glycerol Rich-Phase (Gly)		
	100.w_1_	100.w_2_	100.w_3_	100.w_1_	100.w_2_	100.w_3_	100.w_1_	100.w_2_	100.w_3_
313.15	44.39	44.29	11.32	93.52	2.49	3.99	2.05	80.72	17.23
	41.24	42.02	16.74	89.61	2.90	7.49	2.64	73.28	24.08
	37.78	39.18	23.04	85.70	3.42	10.93	3.21	65.97	30.82
	33.78	35.48	30.74	80.93	3.79	15.28	2.99	56.48	40.53
	29.49	31.11	39.41	76.20	4.02	19.78	2.96	46.91	50.14
	25.72	24.86	49.42	65.59	4.03	30.38	4.92	35.19	59.89
	22.74	20.43	56.83	56.29	5.47	38.24	12.33	25.08	62.59
323.15	43.84	42.89	13.27	85.91	4.61	9.48	1.16	81.63	17.21
	40.65	40.05	19.32	81.65	5.06	13.35	1.73	73.91	24.39
	37.62	37.02	25.36	77.37	5.29	17.34	2.15	66.29	31.56
	35.26	34.63	30.11	73.06	5.72	21.24	2.82	58.62	38.61
	32.21	32.31	35.48	67.81	6.71	25.48	3.01	53.21	43.78
	29.87	30.08	40.05	62.52	7.88	29.61	3.22	47.94	48.84
	27.11	27.63	45.27	56.91	9.31	33.79	4.27	41.72	54.01
	24.97	25.02	50.03	51.52	10.61	37.87	5.31	35.38	59.31

An estimated standard error of 1% was used. ^a^ Standard uncertainties *u* are *u*(T) = 0.5 K, *u*(*P*) = 1 kPa, *u*(*w*) ≤ 1.0 wt%. w_1_: mass FAEE; w_2_: mass fraction glycerol; w_3_: mass fraction ethanol.

**Table 4:** Binodal curve data of the ternary system {FAME (1) + glycerol (2) + methanol (3)} at 313.15 K and 323.15 K at normal atmospheric pressure (101.3 kPa), where *w* is the weight fraction ^a^.

T = 313.15 K			T = 323.15 K		
100.w_1_	100.w_2_	100.w_3_	100.w_1_	100.w_2_	100.w_3_
0.92	83.25	15.83	1.87	83.90	14.23
1.11	79.02	19.30	2.46	77.54	20.00
1.43	58.96	39.61	2.68	58.62	38.70
1.49	68.42	30.09	4.12	47.53	48.35
2.15	48.67	49.18	4.42	28.64	66.94
3.79	38.37	57.84	6.48	18.40	75.13
4.75	27.95	67.31	18.68	8.63	72.69
5.72	18.33	75.96	28.70	4.07	67.24
12.22	8.08	79.71	39.10	3.53	57.37
18.60	4.77	76.63	48.48	2.64	48.89
35.35	1.45	63.20	59.06	1.62	39.32
49.89	1.20	48.91	68.67	1.44	29.89
59.17	0.92	39.91	79.02	0.69	20.29
69.20	0.73	30.07	84.27	0.42	15.31
79.54	0.54	19.92			
85.24	0.31	14.54			

An estimated standard error of 1% was used. ^a^ Standard uncertainties *u* are *u*(T) = 0.5 K, *u*(*P*) = 1 kPa, *u*(*w*) ≤ 1.0 wt%. w_1_: mass FAME; w_2_: mass fraction glycerol; w_3_: mass fraction methanol.

**Table 5:** The phase equilibrium data (liquid + liquid) for the system {FAME (1) + glycerol (2) + methanol (3)} at 313.15 K and 323.15 K at normal atmospheric pressure (101.3 kPa), where *w* is the weight fraction ^a^.

T (K)	Overall Composition (Sol)			Experimental (Tie Lines)					
				Biodiesel Rich-Phase (FAME)			Glycerol Rich-Phase (Gly)		
	100.w_1_	100.w_2_	100.w_3_	100.w_1_	100.w_2_	100.w_3_	100.w_1_	100.w_2_	100.w_3_
313.15	45.98	47.18	6.84	98.99	0.08	0.93	1.28	85.61	13.11
	44.15	44.91	10.94	98.33	0.08	1.59	1.31	79.25	19.45
	42.02	42.34	15.64	97.59	0.08	2.33	1.51	72.87	25.62
	39.46	40.59	19.95	96.92	0.09	3.03	1.69	66.37	31.95
	36.89	37.24	25.87	95.92	0.09	3.99	1.94	59.58	38.48
	34.65	34.82	30.55	95.01	0.09	4.91	2.25	52.66	45.10
	29.30	30.45	40.25	93.24	0.09	6.67	2.80	42.58	54.63
	24.61	24.98	50.42	91.38	0.08	8.54	3.42	33.71	62.87
	20.01	19.76	60.23	89.44	0.08	10.48	3.92	24.89	71.19
323.15	45.36	46.78	7.86	99.26	0.02	0.72	2.03	83.69	14.31
	43.37	44.78	11.85	98.81	0.01	1.18	2.25	77.55	20.20
	41.39	42.88	15.73	98.39	0.01	1.61	2.46	71.53	26.01
	39.63	40.49	19.88	97.78	0.01	2.21	2.72	65.38	31.92
	37.47	37.32	25.21	96.99	0.02	2.99	3.01	59.63	37.37
	34.94	34.94	30.12	96.39	0.01	3.62	3.27	53.76	42.96
	30.81	29.33	39.86	94.14	0.01	5.84	4.04	41.73	54.23
	25.82	24.64	49.54	91.41	0.01	8.58	4.88	32.54	62.58
	21.64	18.63	59.73	88.59	0.02	11.39	5.59	23.42	70.99

An estimated standard error of 1% was used. ^a^ Standard uncertainties *u* are *u*(T) = 0.5 K, *u*(*P*) = 1 kPa, *u*(*w*) ≤ 1.0 wt%. w_1_: mass FAME; w_2_: mass fraction glycerol; w_3_: mass fraction methanol.

**Table 6:** Quality tests for LLE experimental data of the {FAEE (1) + glycerol (2) + ethanol (3)} systems.

T (K)	W^Sol^ kg	Overall Composition			W^Bio^ kg	W^Gly^ kg	W^Sol^ calc kg	δ (%)
		100.w_1_	100.w_2_	100.w_3_				
313.15	0.1543	0.4439	0.4429	0.1132	0.0709	0.0833	0.1542	0.0648
	0.1658	0.4124	0.4202	0.1674	0.0736	0.0922	0.1658	0.0000
	0.1342	0.3778	0.3918	0.2304	0.0564	0.0774	0.1338	0.2981
	0.1739	0.3378	0.3548	0.3074	0.0688	0.1051	0.1739	0.0000
	0.1673	0.2949	0.3110	0.3941	0.0607	0.1067	0.1674	0.0598
	0.1276	0.2572	0.2486	0.4942	0.0436	0.0837	0.1273	0.2351
	0.1428	0.2274	0.2043	0.5683	0.0338	0.1090	0.1428	0.0000
323.15	0.1963	0.4384	0.4289	0.1327	0.0988	0.0975	0.1963	0.0000
	0.1874	0.4065	0.4005	0.1930	0.0914	0.0956	0.1870	0.2134
	0.1953	0.3762	0.3702	0.2536	0.0924	0.1025	0.1949	0.2048
	0.1746	0.3526	0.3463	0.3011	0.0803	0.0944	0.1747	0.0573
	0.1849	0.3221	0.3231	0.3548	0.0833	0.1016	0.1849	0.0000
	0.1841	0.2987	0.3008	0.4005	0.0809	0.1031	0.1840	0.0543
	0.1948	0.2710	0.2763	0.4527	0.0845	0.1103	0.1948	0.0000
	0.1563	0.2497	0.2500	0.5003	0.0664	0.0898	0.1562	0.0640
							δ average:	0.0848

**Table 7:** Quality tests for LLE experimental data of {FAME (1) + glycerol (2) + methanol (3)} systems.

T (K)	W^Sol^ kg	Overall Composition			W^Bio^ kg	W^Gly^ kg	W^Sol^ calc kg	δ (%)
		100.w_1_	100.w_2_	100.w_3_				
313.15	0.1758	0.4598	0.4718	0.0684	0.0804	0.0958	0.1762	0.2275
	0.1684	0.4415	0.4491	0.1094	0.0751	0.0935	0.1686	0.1188
	0.1815	0.4202	0.4234	0.1564	0.0765	0.1052	0.1817	0.1102
	0.1871	0.3946	0.4059	0.1995	0.0742	0.1135	0.1877	0.3207
	0.1963	0.3689	0.3724	0.2587	0.0730	0.1231	0.1961	0.1019
	0.1569	0.3465	0.3480	0.3055	0.0547	0.1022	0.1569	0.0000
	0.1758	0.2930	0.3045	0.4025	0.0515	0.1241	0.1756	0.1138
	0.1637	0.2460	0.2498	0.5042	0.0395	0.1249	0.1644	0.4276
	0.1642	0.2001	0.1976	0.6023	0.0309	0.1339	0.1648	0.3654
323.15	0.1926	0.4536	0.4678	0.0786	0.0858	0.1075	0.1933	0.3634
	0.2041	0.4337	0.4478	0.1185	0.0871	0.1178	0.2049	0.3920
	0.2008	0.4139	0.4288	0.1573	0.0815	0.1199	0.2014	0.2988
	0.1956	0.3963	0.4049	0.1988	0.0759	0.1203	0.1962	0.3067
	0.1839	0.3747	0.3732	0.2521	0.0675	0.1161	0.1836	0.1631
	0.1874	0.3494	0.3494	0.3012	0.0638	0.1234	0.1872	0.1067
	0.1931	0.3081	0.2933	0.3986	0.0574	0.1357	0.1931	0.0000
	0.1868	0.2582	0.2464	0.4954	0.0422	0.1442	0.1864	0.2141
	0.1988	0.2164	0.1863	0.5973	0.0384	0.1608	0.1992	0.2012
							δ average:	0.1910

**Table 8:** Separation factors (S) and distribution coefficients of ethanol (D2) of the {FAEE (1) + glycerol (2) + ethanol (3)} systems.

T (K)	S	D_1_	D_2_
313.15	196.9985	0.0219	4.3183
	109.1209	0.0295	3.2150
	75.4887	0.0375	2.8275
	71.7946	0.0369	2.6525
	65.2561	0.0388	2.5349
	26.2808	0.0750	1.9714
	7.4723	0.2190	1.6368
323.15	134.4492	0.0135	1.8154
	87.7481	0.0208	1.8270
	65.4971	0.0278	1.8201
	47.4192	0.0383	1.8173
	38.7083	0.0444	1.7182
	32.0366	0.0515	1.6500
	21.2995	0.0750	1.5984
	15.1955	0.1031	1.5661

**Table 9:** Separation factors (S) and distribution coefficients of ethanol (D2) in the {FAME (1) + glycerol (2) + methanol (3)} systems.

T (K)	S	D_1_	D_2_
313.15	1090.2093	0.0129	14.0968
	925.2629	0.0132	12.2327
	710.6286	0.0155	10.9957
	610.7680	0.0174	10.6500
	476.8366	0.0202	9.6441
	387.8661	0.0237	9.1853
	272.7405	0.0300	8.1904
	196.7028	0.0374	7.3618
	154.9951	0.0438	6.7929
323.15	986.4360	0.0201	19.8750
	751.7748	0.0228	17.1186
	646.1455	0.0250	16.1553
	518.8951	0.0278	14.4344
	404.0793	0.0309	12.4983
	351.7596	0.0339	11.9333
	216.3812	0.0429	9.2860
	136.6225	0.0534	7.2937
	98.7771	0.0631	6.2327

**Table 10:** Fitted binary interaction parameters of NRTL (α_ij_ = 0.2) model for systems.

System: FAEE (1) + Glycerol (2) + Ethanol (3)					
Temperature (K)	Pair *i-j*	∆g_ij_ (K) ^a^	∆g_ji_ (K) ^a^	rmsd × 100	
				Phase FAEE	Phase Glyc
313.15–323.15	1–2	11.57	2061.04	3.01	1.51
	1–3	−841.28	1845.96		
	2–3	−874.07	2036.37		
**System: FAME (1) + glycerol (2) + methanol (3)**					
**Temperature (K)**	**Pair *i-j***	**∆g_ij_ (K) ^a^**	**∆g_ji_ (K) ^a^**	**rmsd × 100**	
				**Phase FAME**	**Phase Glyc**
313.15–323.23	1–2	2210.27	2357.81	0.93	1.04
	1–3	−648.67	1521.47		
	2–3	−1348.47	−2604.06		

^a^ Fitted parameters were $∆g_{ij}=\left(g_{ij}-g_{ii}\right)/R$.

## Data Availability

The original contributions presented in the study are included in the article and further inquiries can be directed to the corresponding authors.
